# Applications and Developments of Thermal Spray Coatings for the Iron and Steel Industry

**DOI:** 10.3390/ma16020516

**Published:** 2023-01-05

**Authors:** Surinder Singh, Christopher C. Berndt, R. K. Singh Raman, Harpreet Singh, Andrew S. M. Ang

**Affiliations:** 1 Australian Research Council (ARC), Industrial Transformation Training Centre on Surface Engineering for Advanced Materials (SEAM), Swinburne University of Technology, Hawthorn, VIC 3122, Australia; 2 Department of Mechanical and Aerospace Engineering, Monash University, Clayton, VIC 3800, Australia; 3 Department of Chemical Engineering, Monash University, Clayton, VIC 3800, Australia; 4 Department of Mechanical Engineering, Indian Institute of Technology Ropar, Rupnagar 140001, India

**Keywords:** steel making, thermal spray, coating, thermal degradation, wear, high temperature oxidation

## Abstract

The steel making processes involves extreme and harsh operating conditions; hence, the production hardware is exposed to degradation mechanisms under high temperature oxidation, erosion, wear, impact, and corrosive environments. These adverse factors affect the product quality and efficiency of the steel making industry, which contributes to production downtime and maintenance costs. Thermal spray technologies that circumvent surface degradation mechanisms are also attractive for their environmental safety, effectiveness and ease of use. The need of thermal spray coatings and advancement in terms of materials and spray processes are reviewed in this article. Application and development of thermal spray coatings for steel making hardware from the molten metal processing stages such as electric arc and basic oxygen furnaces, through to continuous casting, annealing, and the galvanizing line; to the final shaping process such as cold and hot rolling of the steel strips are highlighted. Specifically, thermal spray feedstock materials and processes that have potential to replace hazardous hard chrome plating are discussed. It is projected that novel coating solutions will be incorporated as awareness and acceptance of thermal spray technology grows in the steel making sectors, which will improve the productivity of the industry.

## 1. Introduction

**Figure 1:**
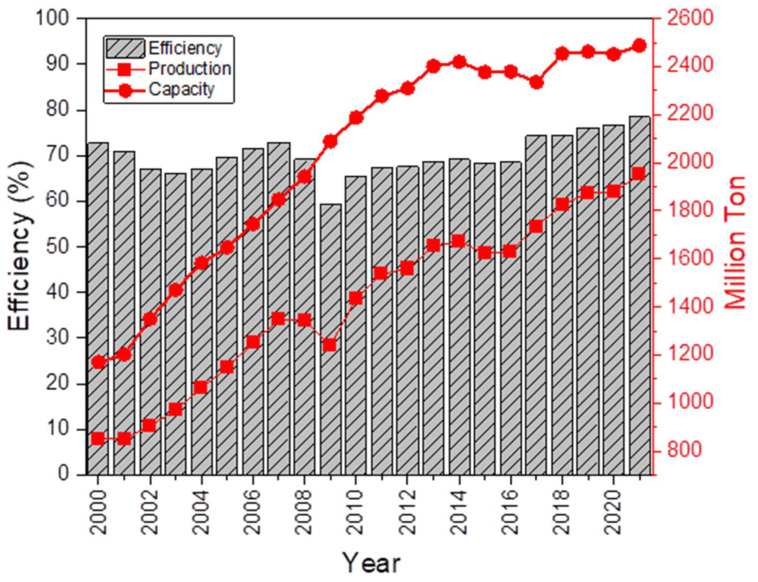
Global annual steel production, production capacity, and efficiency of the iron and steel production system (data till December 2021). Adapted from [1,3,8].

Global steel production increased by 3.7%, from 1880 Mt in 2020 to 1950 Mt in 2021 [1,2]. It has been estimated that post pandemic (COVID-19) global economic recovery will drive stronger consumption of iron ore [3] and global steel production is expected to grow by 32% over the period from 2021 to 2025 in the absence of closures [4]. Additionally, the global steel production capacity has grown by 1.5% from 2452 Mt in 2020 to 2488 Mt in 2021 [2,3]. However, even with the continuous growth of the production capacity, the efficiency of the iron and steel making industry has only a marginal increase (see Figure 1) over the last two decades [5], which highlights technological inefficiencies of the production system. Factors contributing to this marginal efficiency increase include the limitations of the production hardware due to their exposure to extreme and harsh operating conditions such as high temperature, corrosive environment, and erosive wear throughout the steel production process [5,6]. The technically challenging practices within the iron and steel making industry directly influence product quality and efficiency, which contribute to production downtime and greater maintenance costs [7].

The improvement in the surface durability of hardware through implementation of surface modification technologies addresses the gap between actual production and capacity, with the objective of improving product quality, increasing product throughputs, reducing maintenance cost and downtime. Hence, the life of the iron and steel making hardware and the production efficiency [5,7] is enhanced. Improvement in hardware starts with those used for the mineral processing of the raw material and continues with those for the iron and steel making; foundry and casting operations; and shaping and product sizing: all of which is staged in a continuous flow of product.

The extraction of ore and mineral processing is not within the scope of this article, but the surface engineering approaches would be similar for the equipment that are employed for excavation, grinding, crushing and transportation of ores. The raw materials, when delivered to an iron and steel making plant, enter continuous processing that requires tooling to withstand conditions that are defined as ‘harsh and extreme’ where operational hardware is subjected to high temperatures, high pressures, chemical and oxidative corrosion, and wear and erosion [5,6,7]. These technical challenges can be addressed by thermal spray (TS) coatings that protect the equipment from extreme operating conditions [9,10,11]. The surface modifications require hard, wear resistant, high temperature oxidation resistant and thermally stable surfaces. The surfacing process must also be sympathetic to environmental needs [12].

Surface modifications by means of coating technologies protect the substrate material and reduce replacement cost by retarding material degradation, thereby enhancing the service life of components [13]. Coating methods are identified according to specific requirements such as desirable coating thickness; adhesion mechanism and essential mechanical strength; component geometry and chemistry; coating process conditions that are amenable to the component; and operating conditions of the coating [14]. In particular, thermal spray processes are widely proven in industries such as power generation, automotive, aerospace, marine, and petrochemical for protection and repair of components [15].

Thermal spray technology has a favourable history in the steel and other alloy making industries. Moreover, with an increase in steelmaking capacity, hardware components requiring the surface modifications with thermal spraying is also growing. In very broad terms, a thermal spray process involves particles being deposited in a molten or semi-molten state. These particles form splat shaped microstructural artefacts that solidify into a network of interlocked splats and create, typically, a functional coating of 50 µm to several millimeters [16].

**Figure 2:**
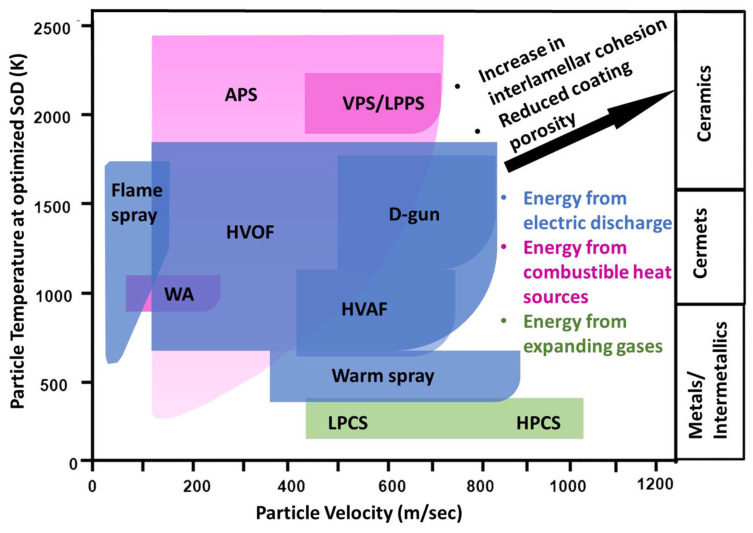
Simplified classification of thermal spray processes according to their source of heat generations, particle velocities and temperatures. APS = atmospheric plasma spray, VPS = vacuum plasma spray, LPPS = low pressure plasma spray, WA = wire arc, HVOF = high velocity oxygen fuel, D-gun = detonation gun, HVAF = high velocity air fuel, LPCS = low pressure cold spray, HPCS = high pressure cold spray, SoD = stand-off distance. Adapted with permission from [16]. 2014 Taylor & Francis.

Figure 2 portrays the broad classification of thermal spray processes based on their prime energy source, particle velocities and temperatures. Thermal spray technology has also proven cost effective and of environmental benefit to enable adoption by the iron and steel industry [5,17,18]. Advancements in materials, equipment and processes are furthering applications in the iron and steel industry.

Developments in coating materials, equipment and thermal spray processes are presented in this article. Application and development of thermal spray coatings in the steel making hardware, from the molten metal processing stages such as electric arc and basic oxygen furnaces, through to continuous casting, annealing, and the galvanizing line, to the final shaping process such as cold and hot rolling of the steel strips are highlighted. Other surfacing technologies such as laser cladding and physical vapor deposition that have demonstrated service in the iron and steel industry are briefly described.

## 2. Thermal Spray Applications in Iron and Steel Industry

**Figure 3:**
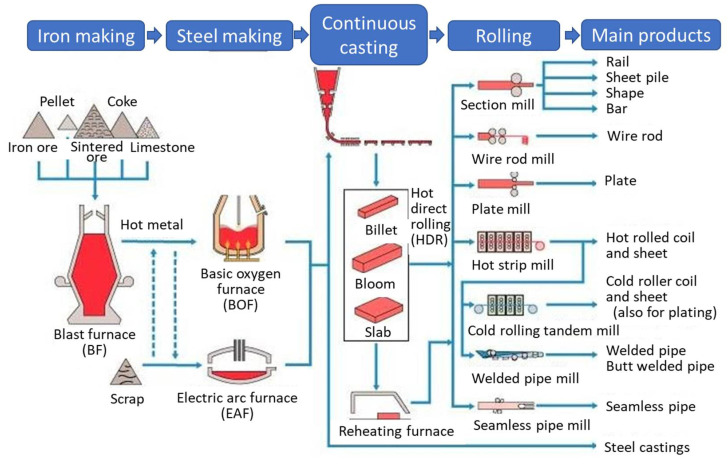
Schematic representation of iron and steel production process consisting of raw material preparation, iron making, steel making, continuous casting and shaping. Thermal spray coatings are employed throughout the manufacturing process. Adapted from [19].

**Figure 4:**
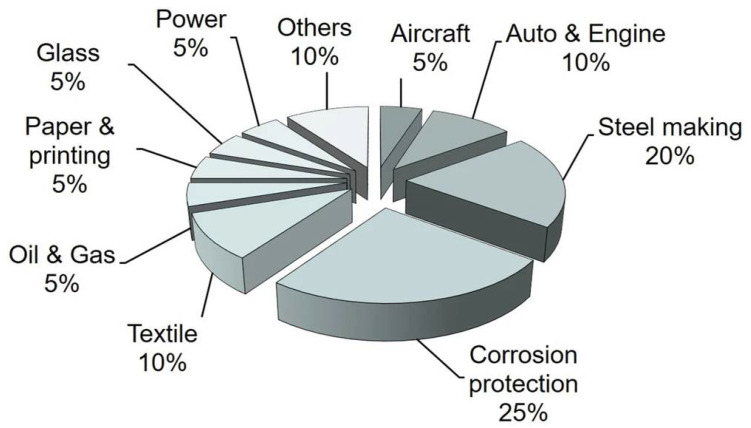
Estimated distribution of thermal spray applications in China in year 2008. Adapted with permission from [20]. 2008, Springer Nature.

Figure 3 [19] shows that steel production integrates five processes: (i) iron making, (ii) steel making, (iii) continuous casting, (iv) rolling, and (v) main products shaping. Surface modification by thermal spray processes plays a major role in steel making, casting, and shaping. In China, thermal spray contributes about a 20% share to industrial applications during the manufacture of steel [20] (Figure 4). Specific uses include components of electric arc and basic oxygen furnaces that degrade due to harsh conditions imposed by thermal shock, thermal cycling, high operating temperatures, and corrosive slag fumes, and gases [21]. Furnace hoods, ducts, gas injecting tuyeres, lances, nozzles, and a multitude of steel processing rolls all experience thermal, mechanical, and chemical degradation [22]. As well, rolls and casters deteriorate and cause production downtime in continuous casting and shaping processes where heat, corrosion and wear are adverse operational environments.

Production equipment benefits from surface modifications that improve life expectancy under the harsh environments of steel production [23]. For instance, wrapper rolls, which coil steel strips, degrade under the high temperatures, wear, high strain, and chemical attack from the hot steel strip. These technical challenges are being addressed by thermal spray coatings that protect the equipment from extreme operating conditions [9,10]. The application of thermal spray coatings for different machinery components of the steel production system is discussed in further sections.

### 2.1. Furnace Hoods and Ducting

**Figure 5:**
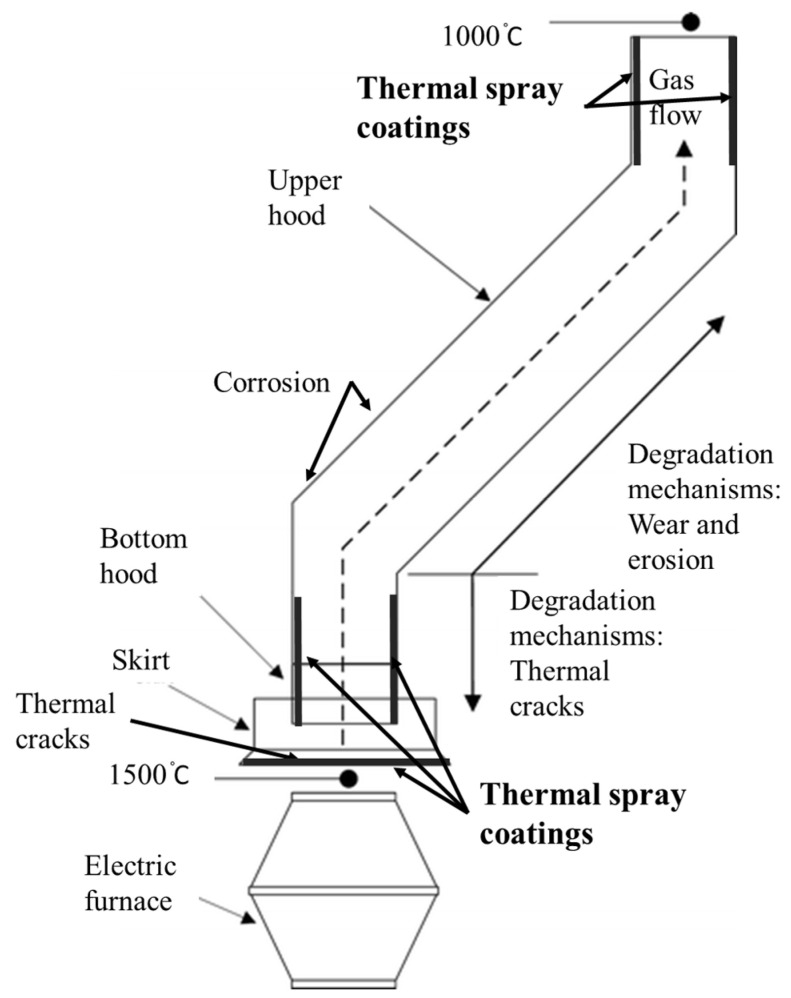
Schematic representation of electric arc furnace gas collecting system and accompanying degradation mechanisms. Reproduced with permission from [5]. 2010, Springer Nature.

Steel production capacity was anticipated to grow by 2–3% through 2021–2022. The major part (72%) of the iron and steel industry comprises tier−1 industries with electric arc and basic oxygen furnaces [24]. The degradation mechanism of gas recovery and ducting systems of these furnaces is indicated in Figure 5 [5,7]. Steel components such as furnace hoods and furnace skirts are water and air cooled to mitigate thermal failures. The hood skirt at the furnace base experiences thermal shock, leading to cracking; while the upper hood is exposed to erosion, wear and high temperature oxidation from hot gases and particle impact [5,25].

Kweon and Kim [25] investigated top coat and bond coat combinations to develop thermal barrier coatings (TBCs) for lower hood and skirt applications using plasma spray technology. Laboratory thermal shock tests and field demonstrations explored the performance of these coatings. Multi-layer coatings that included a Ni-20Cr bond coat of thickness 100 μm, intermediate oxide-based cermet (Al_2_O_3_-30(Ni-20Al), and an oxide top coat of Al_2_O_3_-TiO_2_ sustained longer service times under field testing of two months compared to two-layer (bond and top coat) coatings. Diffusion of silicon underneath the top coat caused spallation of the ceramic, leading to poor interfacial bonding. However, these coatings are a potential alternative to the currently used hard chrome plating.

### 2.2. Gas Injection Tuyeres, Lances, and Nozzles

**Figure 6:**
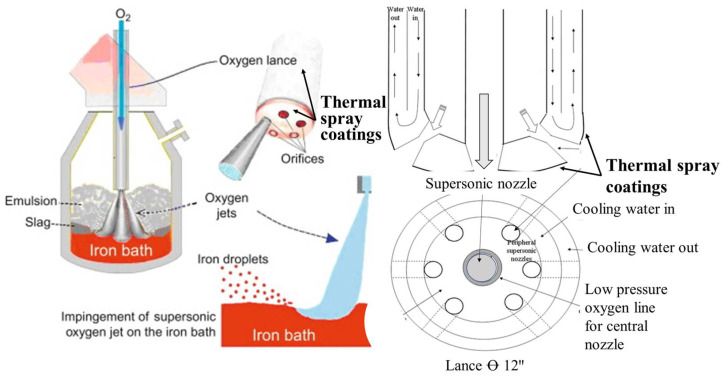
(**left**) Schematic representation of gas/fuel purging system in the furnace and enlarged view of the blowing end of lances that are exposed to the high temperature environment of a blast furnace. Reprinted with permission from [27]. 2014, Elsevier. (**right**) Drawing of the seven-hole lance design with a central subsonic nozzle, adapted from [28]. 2007, Springer Nature.

**Figure 7:**
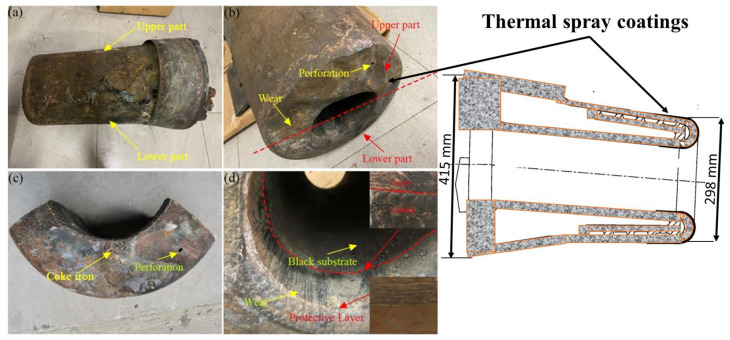
A typical application of thermal spray coatings in protecting blast furnace tuyeres, (**a**,**b**) overall views, (**c**) front end, and (**d**) inner wall of a tuyere of blast furnace, adapted with permission from [29]. 2022, Elsevier.

The tuyere, lances, and nozzles require high thermal conduction for their operational environment and, thus, are manufactured from copper or copper alloy and are water-cooled. A schematic of the gas/fuel purging system in the furnace and an enlarged view of the blowing end of lances that are usually exposed to the extremely high temperature environment of a blast furnace, along with a drawing of the seven-hole lance design with a central subsonic nozzle, is represented in Figure 6. This essential hardware allows the oxygen-air fuel mixture to be blown into a blast furnace [7]. The blow end of tuyeres, lances and nozzles is exposed to the aggressive and harsh environment of the blast furnace and are damaged due to overheating, thermal cycling, and high temperature oxidation; and attack from corrosive gases, molten slag and steel (as represented in Figure 7). This damage causes water leaks that can lead to unsafe explosions and heat losses inside the furnace [5,7,26]. Furthermore, production time and labor is mandatory to maintain damaged tuyeres, lances and nozzles; which all contribute to an adverse operational expense.

Thermal spray coatings have been proposed to protect these components of the blast furnace [23,30]. Nakahira [31] patented a coating system and claimed benefits that provided thermal-shock resistance and high durability to tuyeres. The coating architecture consisted of (i) three consecutive layers of a Ni/Co based self-fluxing alloy, (ii) zirconia or alumina with Ni/Co based self-fluxing alloy, and (iii) pure zirconia or alumina as a final layer. All coating layers employed plasma or oxy-acetylene flame spray technology.

Apte et al. [32] proposed a coating material, identified as MCrAlQ, for protecting tuyeres. ‘M’ signifies an element or a mixture of nickel, cobalt, and iron. ‘Q’ signifies an element alone or a mixture of yttrium, zirconium, hafnium and ytterbium. Additionally, inventors from BAO steel (Song et al. [33]) explored multilayer and multi process coatings. Their design was aimed at improving the thermal load sustainability, hot corrosion/oxidation, and erosion resistance of tuyeres by reducing the mismatch of coefficient of thermal expansions among the substrate, bond coat and top layers. A bond coat of Ni-based alloy was deposited by plasma spray, a middle layer of NiCrAl alloy was deposited by supersonic flame spray (HVOF); and a top layer was a blend of ZrO_2_Y_2_O_3_ and Al_2_O_3_ that was deposited by the plasma spray technique.

Research on coatings for turbines that can sustain high temperature environments is mature [26,34]; but has not addressed specific applications for the iron and steel industry. The technical orientation for these two diverse applications is similar. Thus, R&D on increasing the life span of turbine TBCs [35] can spin off to other areas. Much of this research is focused on reducing the oxidation of the bond coat, from where failure initiates and propagates under thermal cycling [36]. Aluminium was investigated as one of the key elements to minimize the oxide formation at the interface between the ceramic overlay and bond coat. Cyclic oxidation studies were performed with a yttria stabilized zirconia (ZrO_2_-8 wt%Y_2_O_3_, YSZ) top coat. The tests at 1100 °C for 4 h showed superior oxidation resistance in comparison to conventional TBC systems. It was found that durability of the TBCs improves with alumina, which reduces oxygen diffusion owing to its oxide crystal structure and low defect density [37].

A multilayer TBC consisting of a NiCrAlY-20%YSZ-MoSi_2_ top coat has also been investigated since MoSi_2_ prevented diffusion of oxygen that led to bond coat oxidation. Roy et al., 2022 [36] developed a glass ceramic bonded TBC and investigated the thermal cyclic resistance at 1000 °C for 500 cycles No oxide formation and thermally grown oxide layer formation was observed at the bond coat and top coat interface in the glass–ceramic-bonded TBC system. Hence, use of a glass–ceramic bond coat in a TBC system resulted in better heat resistance, oxidation resistance and good stability during thermal cycling compared to conventional TBC systems.

### 2.3. Continuous Casting Moulds

The inlet of the casting machine is a large volume water-cooled mould with an inside lining of copper, the purpose of which is to ensure a continuous flow of steel. Solidification begins near the water-cooled copper lining [38].

**Figure 8:**
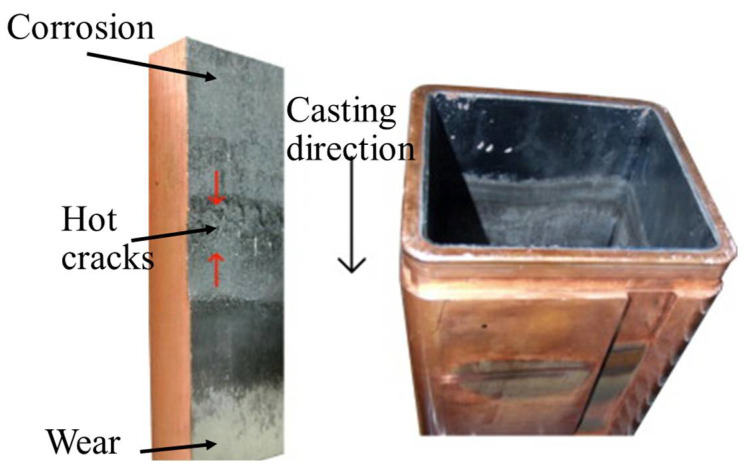
(**left**) Schematic representation of edge wear, hot star crack, narrow surface shrinkage, and high temperature oxidation of copper lining of the mould, and (**right**) Image of mould after use with degraded inner lining. The cross section of rectangular tube moulds typically range from 0.6 m^2^ to 2.5 m^2^. Adapted with permission from [39]. 2014, Elsevier.

**Figure 9:**
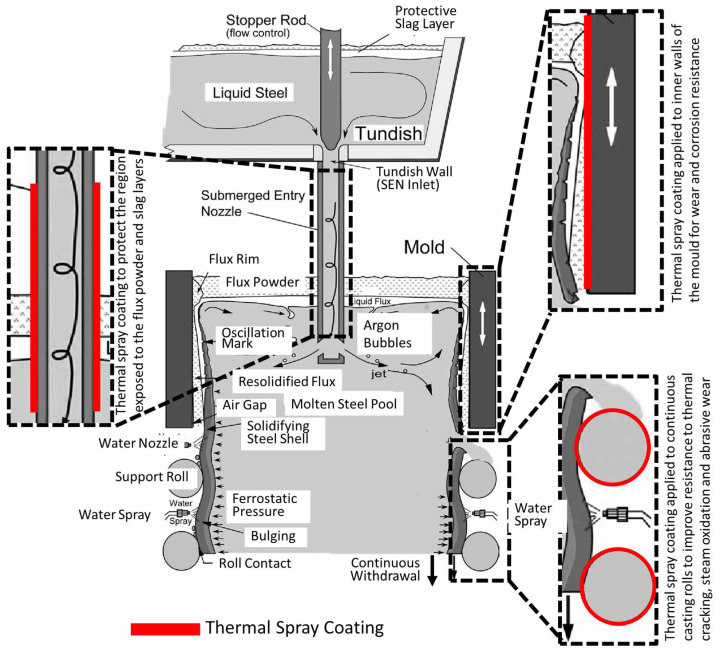
Schematic diagram representing the continuous casting process and potential applications of thermal spray coatings. Reproduced with permission from [43]. 2017, John Wiley and Sons.

The mould is a critical component of the continuous casting setup since this ensures the surface quality of the cast slab and influences the cost and operating rate of the casting production. The copper lining of the mould suffers edge wear, hot star cracking, narrow surface shrinkage, and oxidation as demonstrated in Figure 8 [39]. The solidification rate and shape of the billet is controlled by the copper lining of the mould [38,40,41]. The steel undergoes solidification as it passes through the mould. Therefore, wear of the lower half of the mould becomes prominent [42]. Moreover, diffusion and copper attachment on the solidified steel surface causes ‘star cracking’’ of the steel surface [34]. Thermal spray coatings have been applied on the inner mould surface to protect against the wear and avoid star cracking as represented in Figure 9 [43].

Application of wear resistant, low wettability, high hardness and low-cost coatings on the inside liner of moulds increases their working life. The ‘working life’ refers to the time period through which the mould shows acceptable dimensional stability for steel production [44]. Laser cladding based on TiC/CaF_2_ self-lubricating alloys have been employed for copper lining of moulds. The hardness of the Co-based alloy/20% TiC/10% CaF_2_ (vol.) self-lubricating coating was twice that of a Co-based alloy coating alone. The TiC and CaF_2_ constituents reduced the wear mass rate as well as frictional coefficient due to the uniform dispersion of fine TiC and CaF_2_ particles in a γ-Co matrix.

Development of metal carbide coatings using HVOF on the mould liner, followed by the deposition of an oxide ceramic slurry is a standard coating procedure for protecting the inner surface of moulds. Sanz [41] mentioned that WC-17%Co coatings on the mould inner surface using HVOF spray followed by a top coat mixture of Cr_2_O_3_ +Al_2_O_3_ oxide ceramic is widely adopted. Allcock et al. [40] and Lavin [45] used a chromia-forming sealant as an alternative to oxide slurries and developed a well adhered and highly dense ceramic surface. These coatings resulted in superior friction characteristics compared to electroplated chromium.

Sanz [41] highlighted the effect of ceramic coatings on the thermal conductivity of the mould and suggested coating application only to the most prominent wear zones. However, Lavin [45] specified coating all of the inner surface of the mould with a varying coating thickness. The HVOF sprayed WC-Co coating was thin (0.08 mm) at the top of the mould and increased in a linear fashion to 0.46 mm towards the mould bottom. This strategy protected the mould top from high thermal stresses caused by molten steel as well as the mould bottom from cracking and spallation caused by high pressures instigated by the steel billet.

Table 1 summarises the available data concerning mould linings and submerged entry nozzles (SENs) and other parts of the steel making hardware. The trend of coating materials is changing from alloys to composites that include top layers of ceramics and coating preparation processes of electrodeposition, thermal spray and laser cladding technologies. The hardness, wear, corrosion and oxidation characteristics of the coatings are being improved. Nickel and cobalt are mainly used as base bond coats and Cr, Zr, Si and WC are considered as hard phase materials. Oxides such as Al_2_O_3_, carbides and borides are used as ceramic phases to improve the wear properties. Consideration of nanomaterials is becoming the research focus to achieve better adhesion strength, wear resistance and thermal conductivity of the coatings developed for moulds [45,46].

Electroplated layers of Ni and Cr are widely accepted owing to their wear resistance and negligible effect on mould thermal conductivity. However, the production of hazardous hexavalent chromium compounds during electroplating has encouraged industry to employ thermal spray coatings rather than hard chrome plating [54]. Laser cladding is also being researched to repair the surface of moulds [55]. Thin solid films have been implemented to protect SENs and mould linings [56], but these have limited effectiveness under the highly aggressive industrial conditions.

Carbides are prominently adopted by the industry to optimize the coating materials for mould linings and SENs. Thermal spray coatings are beneficial due to their low production cost, good wear resistance and heat conduction characteristics. Processes such as plasma spray, HVOF, and suspension plasma spray have been used to develop coatings such as WC-Co [48,49], Ni-alloys [57], Ni-alloy with oxide cermet [58], and multilayer coatings of alloys and ceramics [59]. Table 1 summarizes some of these developments.

### 2.4. Submerged Entry Nozzle (SEN)

**Figure 10:**
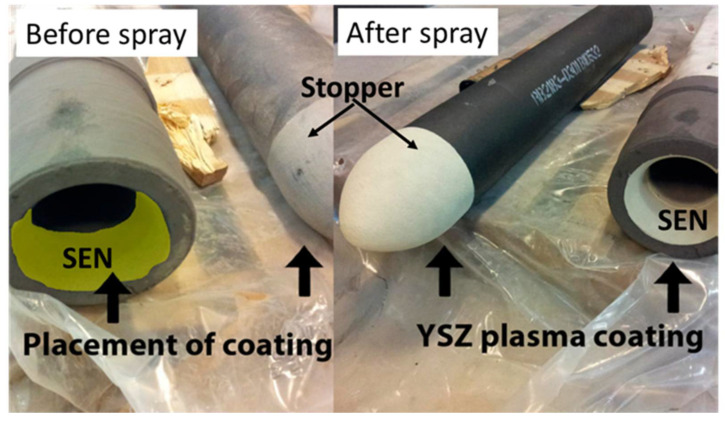
Illustration of a SEN and a stopper rod coated with YSZ by plasma spraying. SEN = submerged entry nozzle, YSZ = yttria stabilized zirconia. The approximate diameter of this nozzle is 185 mm. Adapted with permission from [23]. 2018, Taylor & Francis.

Molten steel is poured from the furnace into a ladle and then into the tundish, from where it enters the continuous casting machine through a shroud that is termed as the submerged entry nozzle (SEN) [29]. The outer SEN lining is thermal spray coated to avoid damage due to molten steel slag and to prevent clogging, as represented in Figure 9 and Figure 10 [23,43]. Svensson et al. [23] developed atmospheric plasma spray yttria-stabilised zirconia (YSZ) coatings for nozzles, SENs and stoppers to prevent clogging during continuous casting of steels. The total mass of the steel teemed through the nozzles increased with a YSZ coated nozzle and the tendency for clogging of the nozzle was reduced, with the nozzle clogging factor being reduced from 0.44 to 0.15. In addition, Memarpour et al. [30] developed glass coatings for SENs and improved casting performance in the casting process.

Smola et al. [54] found that a 1 µm thick layer of Cr–Ni coated by magnetron sputtering physical vapor deposition (MSPVD) increased the resistance of steels to thermal shock because of the formation of chromium-rich oxides such as Cr_2_O_3_, NiCr_2_O_4_, and MnCr_2_O_4_.

### 2.5. Caster Rolls

**Figure 11:**
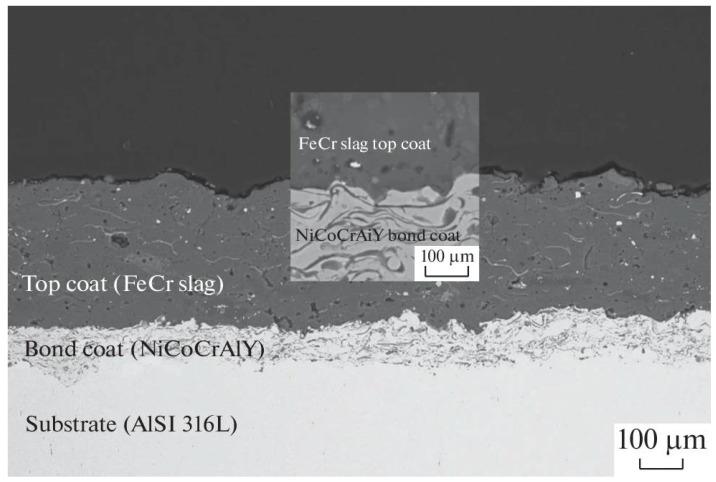
Cross-sectional SEM image of FeCr slag coating as an alternative coating material for caster rolls a continuous casting line. Adapted with permission from [9]. 2022, Springer Nature.

The solidifying steel exits the continuous casting mould through a set of retaining rolls, termed as the ‘caster rolls’, that provide dimensional stability to the cast shape, Figure 11. The rollers redirect the steel from a vertical position to a horizontal bed, where the steel is further cut into slabs, billets or blooms, as shown in Figure 3 [3]. The caster rolls are exposed to steady state temperatures of around 500 °C, with thermal loads and thermal cycling due to inevitable stoppages in the continuous casting operation [51]. Additionally, the weight of the steel strand and pressure head from the molten steel causes heavy stresses at the caster rolls [42]. Furthermore, the water spray that cools and lubricates the rolls initiate oxidation of the rollers and steel that causes thermal cracking at the roller surface [5,7]. These operational factors, in addition to the abrasive wear on roller surfaces caused by steel oxides, casting slag, and mineral deposits, result in reduced roll diameters. Reduction of the roller diameter affects the dimensional accuracy of the steel product [60]. Therefore, caster rolls are protected with coatings to improve their performance.

Welding, thermal spray and laser cladding have been implemented to develop coatings on caster rollers [42,56]. HVOF, detonation gun, plasma spray and laser cladding technologies have been used to deposit carbides and oxide layers on the caster rolls. Ju et al. [61] improved the wear, high temperature oxidation and hardness of the rollers by depositing Fe-based powders and 42CrMo steel (also designated as 4140 or 42CrMo4) using laser cladding technology. Laser cladding parameters were optimized so that the weight loss of the roller was reduced to half of the weight loss of the bare substrate, and the hardness of the coated surface was improved three times that of the original substrate. Similarly, Makarov et al. [62] reconditioned rollers by developing Fe-25Cr5VMoSi (equivalent of PP-Np-25H5FMS) and Fe-Co-Cr-Mo based coatings using wire arc and laser cladding technologies, respectively. Laser clad surfaces showed 4.95 times lesser wear rate compared to wire arc coated surfaces owing to the ultra-high cooling rates in laser cladding that gave rise to harder phases.

Wang et al. [63,64] developed Cr_3_C_2_-25NiCr coatings using detonation spraying. It was found that fatigue cracks, also described as alligator or crocodile cracks, were formed on uncoated rollers after 3740 heating-cooling cycles. No such cracks were observed in coated rolls after 12,000 cycles. Sun et al. [65] improved the wear and thermal shock resistance of rollers by developing a WC-20Cr-7Ni coating using plasma spray technology.

### 2.6. Wrapper and Process Rolls

**Figure 12:**
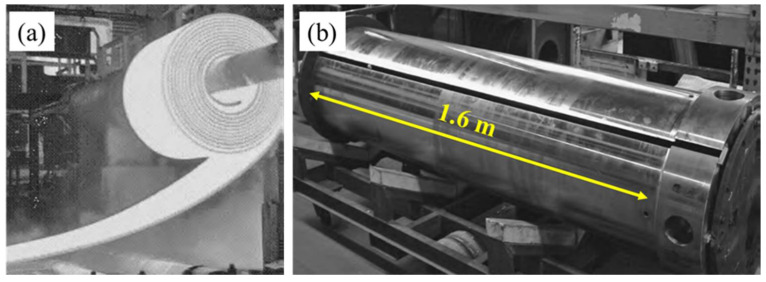
(**a**) Hot steel coil being wound, showing the harsh conditions of steel manufacturing. (**b**) Image of a wrapper roll. Adapted with permission from [7]. 2013, ASM International.

Wrapper rolls are used to wind the hot steel strip into a coil as shown in Figure 12. Wrapper rolls experience a harsh environment because the hot steel strip is forced to turn around itself to form a coil. Heavy stresses, frictional forces, wear by surface oxides produced due to water spray cooling of the steel strips and from slag materials, and heat loads all act simultaneously on the wrapper rolls during the coiling operation [42]. Hard oxide particles and slag deposits become embedded into the hot strip, causing damage [65,66]. Few coatings can sustain this harsh environment, with nickel-based self-fluxing alloys that are combustion sprayed being among the suitable coatings. Additionally, submerged arc welded coatings can be applied for such applications. Carbide coatings produced by detonation gun and HVOF methods [7] confer surfaces that are harder than the cast strip materials. The surface profiles still require optimization so that there is sufficient friction to grip the strip over the roller without affecting the surface finish of the steel strip.

After casting, the steel is processed further through hot rolling mills to produce slabs, sheets and blooms. At each stage, rollers, such as bridle and furnace rolls, process the steel. Surface properties of hardness, wear and high temperature oxidation determine the quality and surface finish of the steel product. Surface coatings are applied by thermal spray processes to develop specific surface functionalities so that the rolls can operate under these harsh operating environments [67,68]. Chromium has been considered for bridle rolls owing to its surface profile of domed grains, which provides an optimum surface roughness to prevent slippage without degrading the surface properties of the bridle rolls. Additionally, there has been growth in developing cermet coatings such as WC-CoCr, WC-NiCr, and NiCr-Cr_3_C_2_, by using HVOF. HVOF has replaced the hard chrome plated coatings for application in bridle roll coatings owing to the better wear and high temperature oxidation resistance properties of HVOF sprayed cermet coatings along with the enhanced environmental and occupational health and safety benefits [47]. These coatings have 4 to 5 times higher abrasion wear resistance than hard chrome [69].

### 2.7. Annealing Line Rolls

**Figure 13:**
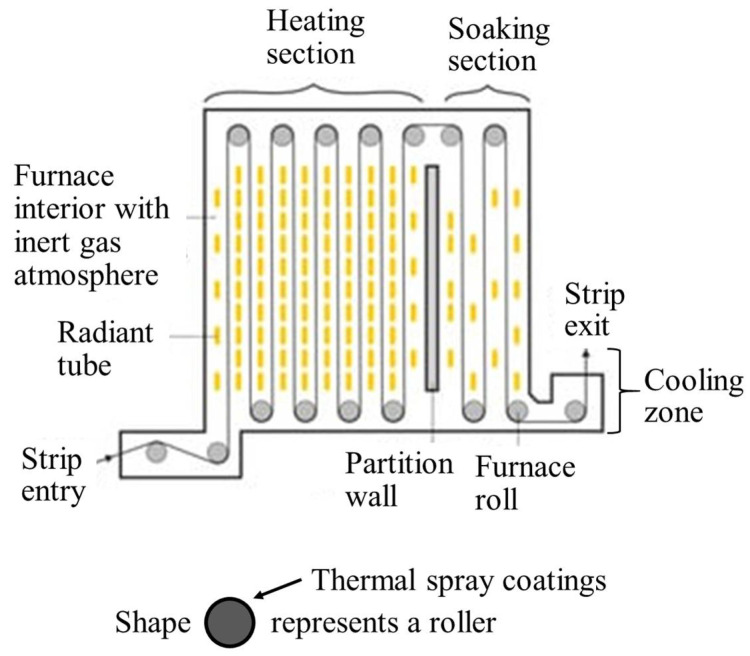
Different heating zones of annealing line. Adapted from [72].

Cold rolled steel slabs and sheets are further processed in annealing rolls to improve ductility. For most steels, annealing is performed in the temperature range of 730–830 °C in a reducing environment [5,70]. Annealing is performed in multiple zones, starting from a rapid heating zone and a soaking zone, followed by cooling zones (Figure 13). The material is exposed to thermal cycling loads. The furnace rolls should be oxidation-resistant under high temperature reducing environments, have resistance to thermal cycling, and maintain its surface roughness to avoid strip slippage [71].

Thermal spray processes such as HVOF or detonation gun have developed coatings for annealing line rolls. The most common limitation in annealing line rolls is oxide build up during mill operation. Huang [58] developed a HVOF sprayed CoCrAlY-CrB_2_-Y_2_O_3_ coating. The coatings were tested in a simulated environment to produce Fe and Mn oxides in a furnace at 900 °C with inert gas purging. Manganese rich oxides were observed to build up on the coating when a Mn-rich steel was the substrate; whereas such oxide build up was much-diminished when a Mn-poor steel was used. It was predicted that Mn enhanced the oxide build up due to the formation of chromium and aluminium oxides on the cermet coatings. The latter oxides react with the manganese of the steel and reduced the service life of the coatings on the rolls. These coating chemistries are being further optimized with investigations on the addition of alternative oxides and/or carbides.

Yu et al. [51] demonstrated that cermet coatings with lower chromium concentration and without Cr-containing carbides or borides resulted in the best resistance to manganese build up. The authors showed that a NiCrAlY-Y_2_O_3_ cermet coating resists Mn build up best in comparison to CoCrAlY-Y_2_O_3_-CrB_2_, CoNiCrAlYZr-Cr_3_C_2_-ZrB_2_ and CoNiCrAlY-Cr_3_C_2_-Y_2_O_3_ HVOF sprayed coatings. The cermet coatings based on NiCrAlY are used preferably for the high temperature zone of the annealing line rolls. However, Cr rich coatings such as NiCr-Cr_3_C_2_ are used for the low temperature heating zones and furnaces that anneal low manganese steels.

### 2.8. Continuous Galvanizing Line

**Figure 14:**
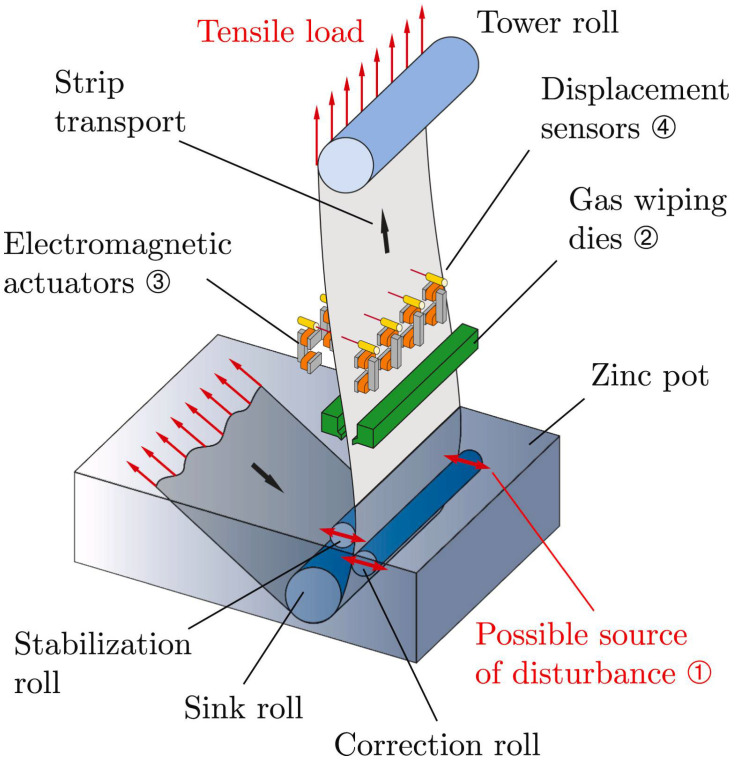
Schematic representation of hot dip galvanising process. Reprinted with permission from [73]. 2020, Elsevier.

Annealed steel sheets are generally coated with a zinc-based coating applied by the hot-dip galvanize process (Figure 14) to improve high temperature oxidation resistance in outdoor environments [73]. Commonly, the applied zinc-based coating comprises zinc with <0.3 wt% Al, Zn-5 wt% Al, or Zn-55 wt% Al, and the bath temperatures are 445–455, 425 and 600 °C, respectively [6]. Different rollers such as the sink roll, stabilizer roll, and correcting roll are used in the galvanizing line to process the steel through the hot dip zinc bath. Additionally, chemical corrosion of the galvanizing line equipment that is in regular contact with the molten zinc or zinc alloy reduces the equipment life. Specifically, the lifespan of roller bearings is limited to 6–30 days depending on the operating environment. Corrosion of the roller bearing, roller surfaces and other submerged parts degrades the final surface quality of the steel strips. Degradation is caused by the reaction of Zn and Al with the iron of the steel rolls. Some of the reactions produce dross particles comprised of FeZn_7_ and Fe_2_Al_5_Zn_x_ (x = 4.5–5.5). These dross particles affect the surface finish of the steel strips due to their higher hardness than the steel strip and, as well, influence the quality of zinc alloy coating [6].

HVOF and detonation gun are being investigated for deposition of WC-Co cermet coatings on galvanizing line hardware owing to the excellent high temperature oxidation resistance of WC-Co alloys compared to Fe and Co alloys [58]. The efficiency of the cermet coatings relies on the spray parameters, feedstock type and whether any surface modifying agent such as a sealant is employed. In contrast to the traditional WC-Co wear resistant coatings, sintered/crushed and plasma densified powders produce WC-Co, W_3_CCo_3_, W_6_CCo_6_, W_6_C_2.54_ and W_4_CCo_2_ that are resistant to zinc oxidation. Decarburization is often associated with the thermal spraying of WC-Co that produces W_2_C; which induces brittleness in the coating [47]. However, decarburization in coatings for galvanizing line hardware improves the zinc oxidation resistance by improving the fraction of Co_3_W_3_C and Co_6_W_6_C carbides. An increase in carbide concentration leads to a decrease in free Co content. The key to increased life is to reduce the amount of free Co in the thermal spray coated cermets. Hence, thermal spray cermet coatings can be developed successfully with decarburized WC-Co feedstock. The effect can be further enhanced with the use of finer carbide feedstock. Moreover, addition of Cr to the Co binder results in the formation of a Cr_2_O_3_ layer. The molten Zn-Al alloy does not wet the Cr_2_O_3_ layer and, hence, the formation of dross particles is prevented [74].

**Figure 15:**
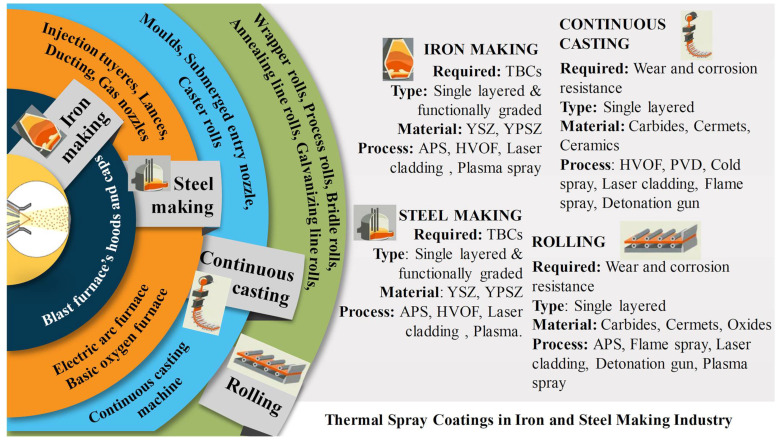
Demonstration of use of thermal spray coatings for various components of iron and steel making industry. APS = atmospheric plasma spray, HVOF = high velocity oxygen fuel, PVD = physical vapor deposition, YSZ = Yttria stabilized zirconia, YPSZ = Yttria partially stabilized zirconia.

Takatani and Kobavashi [75] found that thick (200 μm) HVOF coated WC-Co coatings, with spray dried feedstock, dissolved in a zinc bath within 48 h. However, negligible thickness loss was observed after 96 h for a sintered/crushed feedstock and the same HVOF process. Similarly, Jarosinski et al. [76] demonstrated that the corrosion and oxidation resistance of WC-Co HVOF sprayed coatings improves on reducing the carbide grain size from 3 μm to 1 μm, on keeping the cermet composition fixed. The authors found that, oxides such as YSZ and Al_2_O_3_ are potential corrosion and oxidation resistant coatings for the submerged hardware of the galvanizing line due to their minimal wetting by molten zinc. Furthermore, multilayer coatings with Co- and Mo-based bond coats (e.g., CoCr and MoB), a middle layer of oxide-based ceramics (Al_2_O_3_-TiO_2_), and a top coat of YSZ are an alternate solution to the thin and short life oxide coatings. The multilayer coatings have significantly higher resistance to dissolution than the WC-Co and WC-CoCr coatings in Galvalume^®^ baths due to the CoCrMoB phase formation. (Galvalume^®^ is proprietary steel with a coating consisting of 55 wt% aluminium, 43.4 wt% zinc and 1.6 wt% silicon over the base metal). Figure 15 represents a summary of TS coating methods, materials, and coating routes that have been implemented for various types of hardware of the iron and steel making industry.

## 3. Growth Opportunities for Thermal Spray in the Iron and Steel Industry

### 3.1. Hard Chrome Replacement with Thermal Spray

Surface modifications require hard, wear resistant, high temperature oxidation resistant, and thermally stable surfaces [12]. The surfacing process must also be sympathetic to environmental needs. Hard chrome electroplating is being used to protect iron and steel making machinery from adverse operating environments. Hard chrome electroplating produces hazardous hexavalent chromium compounds [77,78] that have compelled industry to switch to environmentally friendly processes such as thermal spray technology [12,77,79]. High velocity air fuel (HVAF) spray, laser cladding, and high velocity oxygen fuel (HVOF) spray are alternatives to hard chrome plating [12,46,79] that also confer the required coating attributes of wear resistance, high hardness and high temperature oxidation resistance. The fuel source in HVOF is oxygen from a gas or liquid fuel; compressed air is the fuel source in HVAF and electrical energy is used in laser based processes. Thus, unlike electroplating, no hazardous solvents or chemicals are used, since the feedstocks are alloys or ceramics in powder or wire form [80].

Manufacturing industries perceive advantages in exploring thermal spray processes that can form coatings from a diverse array of technically viable feedstocks to replace hazardous hard chrome plating. In this regard, Schroeder and Unger [77] demonstrated the potential of thermal spray coatings by developing WC and CrC coatings using plasma spraying. The authors have produced carbide coated coupons with equivalent properties to hard chrome plating. Sartwell et al. [78,79,81] developed WC/17Co and WC/10Co4Cr HVOF coatings that demonstrated superior performance than hard chrome plating in terms of fatigue, corrosion, wear, impact, hydrogen embrittlement, and cost analysis. In the last decade, R & D efforts in terms of materials and thermal spray process optimisation have been made to replace the hard chrome coatings with thermal spraying, which are presented in Table 2.

### 3.2. Thermal Barrier Coatings

Thermal barrier coatings (TBCs) have an extensive history in the turbine industry [32,87,88] where the operating regime is about 1100–1200 °C in a gaseous environment. There has also been significant development of TBCs for boiler tubes that operate around 800 °C; especially within the coal-fired energy production sector [26,34]. However, there has been limited development of TBCs for steel making furnaces that are required to sustain 1000–1400 °C. Development of TBCs for turbine applications has been vastly explored by researchers [9,10,89,90]. Implementation of these known and well-characterised TBCs for the iron and steel making industry, with additional optimisation for these different operational conditions, would facilitate hardware refurbishments and improve plant efficiencies.

**Figure 16:**
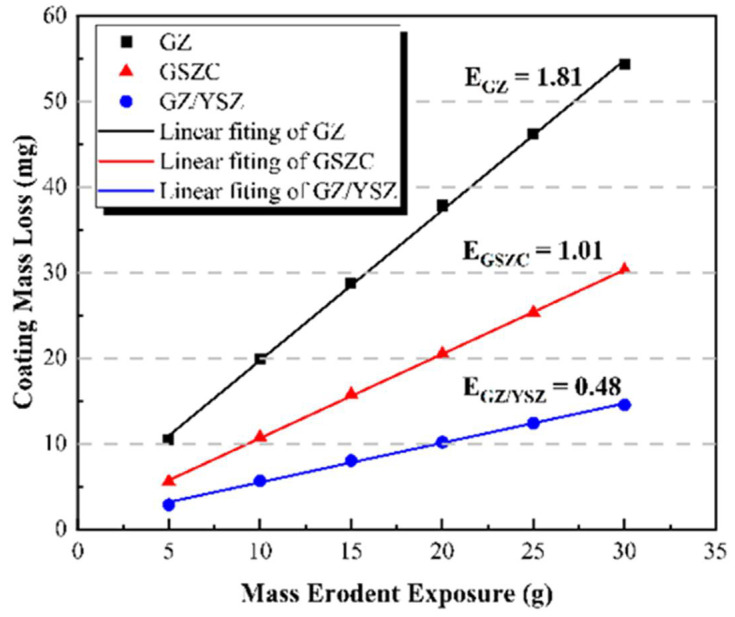
Coating mass loss as a function of erodent exposure for gadolinium zirconate (GZ), gadolinium zirconate and yttria stabilized zirconia (GZ/YSZ), and gadolinium-zirconate-blended feedstock (GSZC) thermal barrier coatings. Reprinted from [91].

Yang et al. [91] in 2021 developed a two layered toughened gadolinium zirconate (GZ) TBC using the atmospheric plasma spray (APS) process. Three coatings, (i) GZ, (ii) GZ/YSZ prepared by blending Gd_2_Zr_2_O_7_ and YSZ powders, and (iii) GSZC prepared by blending (Gd_0.925_Sc_0.075_)_2_(Zr_0.7_Ce_0.3_)_2_O_7_ powders were tested for thermal shock resistance between 900 °C and 1450 °C. The simple GZ coatings sustained 38 thermal shock cycles compared to GZ/YSZ at 33 cycles and GSZC for 7 cycles. On the other hand, the GZ coating exhibited the highest erosion rate of 1.81 mg/g whereas the GZ/YSZ-blended feedstock resulted in the lowest erosion rate of 0.48 mg/g (Figure 16).

A multilayer TBC consisting of a NiCrAlY-20%YSZ-MoSi_2_ topcoat has also been investigated since MoSi_2_ prevented diffusion of oxygen that led to bond coat oxidation. Roy et al., 2022 [71] developed a glass ceramic bonded TBC and investigated the thermal cyclic resistance at 1000 °C for 500 cycles No oxide formation and thermally grown oxide layer formation was observed at the bond coat and top coat interface in the glass–ceramic-bonded TBC system. Hence, use of glass–ceramic bond coat in a TBC system resulted in better heat resistance, oxidation resistance, and good stability during thermal cycling compared to conventional TBC systems. Moreover, Ang et al. [11] have reported that an adhesion strength of around 80 MPa is achievable for the HVOF sprayed ceramics and 50 MPa for atmospheric plasma sprayed ceramics. Therefore, HVOF is most appropriate for bond coats and APS to melt oxide ceramics to establish a top coat for developing functionally graded TBCs.

Gao et al. [92] indicated that YSZ was not suitable for long term exposures above 1200 °C due to phase transformations and sintering. The microstructural changes reduce strain tolerances that result in coating failure. Gao et al. [93] in 2021 proposed Gd_2_O_3_ doped lanthanum cerium oxide (La_2_Ce_2_O_7_, ‘LC’) as a candidate TBC owing to its low thermal conductivity and good phase stability. Further optimization in composition and microstructure, powder composition and spray parameters are warranted. Table 3 reports developments concerning the thermal spray process and TBCs for the steel making process.

### 3.3. Foam Filters

Foam filters are being developed to decrease casting defects in steel strips that are induced while pouring the molten material from the furnace to the ladle or continuous casting machine inlet [96]. Ceramic foam filters such as silicon carbide, zirconia, and carbon-bonded alumina filters are currently being used by the steel making industry. Additionally, surface coatings of materials such as multi-walled carbon nanotubes (MWCNTs) incorporated into the thermal spray coating and flame-sprayed alumina coatings have been applied onto foam filters to improve their service life and performance. Further research in terms of coating materials and methods is needed to maximize the service life of the improved foam filters [96,97].

### 3.4. Pipe Manufacturing

Steel strips pre-coated with zinc coating (galvanised), aluminium coating (aluminised) or Zn/Al alloy coating (Galvalume^®^, Zincalume^®^, Aluzinc^®^, Zalutite^®^, and Galfan^®^) [5,7] are used to manufacture corrosion resistance tubes. Tube is produced by electrical resistance welding of the steel sheets [98]. The heat generated around the weld area and the subsequent tooling operation to remove the weld fin spoils the external pre-coating around the weld area. This weld seam is a preferential site for corrosion, thus; unless protected, this area of the tube will corrode. The weldment can be metal sprayed, for instance, by using the two-wire arc process. This “in-line” method of manufacture is the only method available that offers the flexibility of producing tube with zinc, aluminium or Zn/Al coatings [98].

### 3.5. Roller Bearings

The bearings of annealing or galvanizing line rolls operate under adverse conditions, such as temperature cycles, dust, steam, water and pollutants. Roller bearings performing under high-speed rotation and heavy loads are required to offer high precision and reliability [99]. The harsh operating environment affects the surface conditions of the taper roller of the bearing. High temperature oxidation on the surface of the taper rollers of the bearing increases the friction between rollers and results in non-uniform movement of the steel strips over the rolls, which influences the surface quality of the steel strip. The service life of the bearings would be enhanced by surface engineering that would also improve the quality of steel strips. Conventional thick thermal spray coatings would not be the process of choice. However, thin coatings applied by liquid thermal spray technology [44,100,101] are potential process methods for this application. As well, thin PVD and CVD coatings that have a high surface finish would be viable as wear, corrosion, and oxidation resistant materials [102,103].

### 3.6. New Material and Process Developments

Researchers are developing high entropy alloy (HEA) bond coats that can better sustain thermal shocks [104,105,106]. Meghwal [107] has developed HEA thermal spray bond coats for extreme engineering environments such as turbine engines and boilers. The author has primarily explored the Cr_3_C_2_-NiCr based HVOF sprayed HEA coatings for application in steam turbines due to their oxidation-resistant characteristics. Further development of HEAs holds merit as a coating material for steel making furnaces. Moreover, developments in MoB/CoCr coatings showed significant improvement in corrosion resistance compared to conventional WC-Co and WC-Co-Cr coatings in high Al-content galvalume baths [6]. As well, coatings that are wear and oxidation resistant at high temperatures are development opportunities. There are on-going investigations to develop new materials and processes for the molten galvanizing tub, such as Zn-Mg-Al coating (Magnelis^®^), and Zn-Mg-Al coating (Zagnelis^®^) [108], which provide opportunities for coating hardware that is submerged in these tubs. A recently developed technology, Jet Vapor Deposition (JVD) [108], has produced a zinc coating (Jetgal^®^) onto steel strips to improve their longevity, which has emerged as a potential coating alternative for galvanizing lines [108].

Similarly, a recently (2022) patented Arc Voltage Drive (AVD) system has the capability to develop high-density coatings by automatically controlling the motor speed to maintain a perfect arc gap [109]. This twin wire arc spray system is claimed as having higher productivity and being more economical than other thermal spray processes [109]. Moreover, non-skid coatings have been produced using specifically made materials (SafTrax^®^) [109]. These non-skid coatings can be applied onto iron and steel making equipment such as the furnace, wrapper, and continuous casting rolls. In addition to slip resistance, these coatings resist corrosion, wear, and abrasion. These coatings have a projected lifespan of more than 10 years [109].

**Figure 17:**
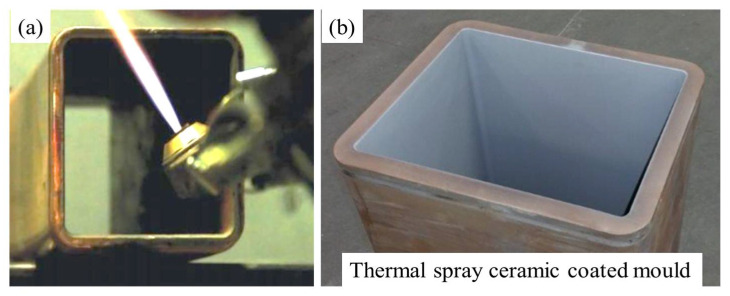
(**a**) HVOF spray torch coating inner walls of a small copper mould, (**b**) Thermal spray ceramic coated rectangular tube copper mould [111]. The approximate inside cross-sectional dimensions of the mould are 180 mm × 130 mm.

New generation TS torches have the capability to coat inner walls of small structures with minimum recommended internal dimensions of 63.5 mm [110]. The development of small TS torches allows coating of the complex geometries that are present in the hardware system of a steel plant [111]. These torches have been used to develop novel ceramic coatings for moulds with higher thermal conductivity, higher hardness, low friction, and superior surface finish compared to conventional TS coatings. Such coating properties provide extended mould service life, better cast product quality, and reduced caster operating costs, while maintaining the integrity of the original copper mould [111]. A typical example of TS ceramic coated mould is shown in Figure 17.

An implementation of TS ceramic coatings to improve compression resistance has been reported for metal seated ball valve applications [112,113,114]. These ceramic coatings have improved the load carrying capacity and tribological performance of the base material, leading to extended service life of hydrometallurgy components. In this regard, Vernhes et al. [113] developed nanostructured Cr_2_O_3_, *n*-TiO_2_, and *n*-TiO_2_-Cr_2_O_3_ TS coatings for metal seated ball valve applications using HVOF spraying and tested the performance of each material’s coating. The results indicated that *n*-TiO_2_-Cr_2_O_3_ coating showed best overall tribological performance compared Cr_2_O_3_ and *n*-TiO_2_ coatings. Specifically, a twofold increase in abrasion resistance was observed for *n*-TiO_2_-Cr_2_O_3_ coatings compared to two other coatings. The steel industry has similar operational challenges as this ball valve application. Thus, these nanostructured material coatings and technologies might be adopted for the iron and steel making industry.

## 4. Concluding Remarks

**Figure 18:**
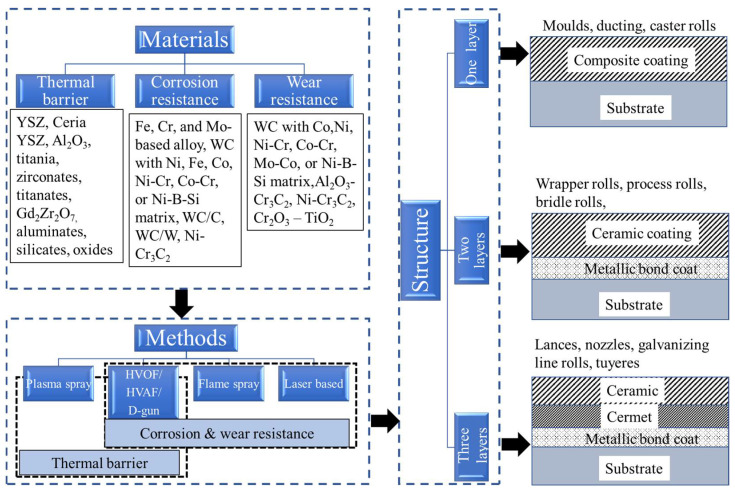
A schematic representing the materials, methods and structure of the coatings that are being applied in iron and steel making industry.

The iron and steel industry is often regarded as a fully mature industry using proven processes with only incremental technological developments. In the context of developments, the thermal spray (TS) industry promises scientific and technological advances to provide solutions to the challenges of the modern iron and steel industry. For instance, this is reflected in the development of tailored multilayer corrosion resistant coatings for the hardware used in galvanizing baths. Thermal spray is available to replace the environmentally hazardous hard chrome plating process. TS technologies are considered as environmentally safe, effective, and easy to apply. Implementation of TS in iron and steel making industries can fulfil the global goal towards green manufacturing. Currently, thermal spray processes such as HVOF, HVAF, APS, flame spray and fuse, and D-gun are widely used for coatings to benefit steel making hardware. HVOF and HVAF spray are employed for developing wear and corrosion resistance coatings of cermets, with laser cladding as another possibility. APS is mainly used for developing ceramic based thermal barrier coatings. A concluding schematic representing the materials, methods and structure of the coatings being applied in iron and steel making industry is shown in Figure 18.

Further advancements in terms of feedstock materials and deposition techniques are ongoing to minimize the gap between production capacity and actual steel production by minimizing downtime and costs. As industry awareness and acceptance of thermal spray technology continues to develop, improved and innovative coating solutions will be increasingly adopted as routine operational practice. For instance, cold spray could be a strong competitor to previous TS processes in terms of producing high quality coatings for corrosion and wear resistance. On-site repair capabilities of TS should also be explored in the steel industry, which can further enhance economic and productivity benefits. Moreover, robots are mainly used in TS processes to control the spray torch movement for creating complex geometries and assure safe working environment. The integration of robotic automation provides flexibility and reproducibility to the manufacturing process and leads to high quality and low-cost coatings [115,116]. Hence, integrated automation with TS processes provides faster onsite repair of the damaged components, which leads to increased productivity of the steel making industry by reducing production down time.

Thermal spray technologies shall continue to contribute significantly to the productivity of the current steel making process chain. Thermal spray solutions will enable significant advancements in the next generation of equipment through improved surface functionality, durability and reliability.

## Figures and Tables

**Table 1:** Coating composition and process used to develop protective coatings for hardware of an iron and steel making plant.

Preparation Method	Coating Composition	Hardness (HV)	Properties	Application	References
HVOF	WC-12Co	1350–1450	Coefficient of friction (CoF = 0.5–0.8)	SEN	[47]
Supersonic flame spraying	WC-10Co-4Cr		Adhesion strength 80 MPa		
Laser cladding	WC-12Co	1350	Good adhesion strength (60 MPa)	SEN	[48]
Laser cladding	Co-based alloy/TiC/CaF_2_ self-lubricating composite	500–1120	CoF < 0.20 at 3000 m distance, Wear rate < 0.8 × 10^−5^ g/Nm	Continuous casting mould	[44]
Chemical slurry with flame spray + heating	WC-Co	1500	Excellent wear resistance	Continuous casting rollers and mould	[45]
APS	NiCr with varied thickness near edges	-	High wear and corrosion resistance	Continuous casting machine mould	[46]
APS	Al_2_O_3-_Cr_3_C_2_	580	Better wear resistance than other Cr based materials	-	[41]
Cold spray	Nanosized WC-Co	1100	CoF = 0.471, low wear rate	-	[49]
APS	65%(NiCrSiFeBC)–35%(WC–Co)	1000	Adhesion strength is 45 MPa. CoF = 0.35–0.43	-	[50]
HVOF	NiCrAlY-Y_2_O_3_, CoCrAlY-Y_2_O_3_-CrB_2_, CoNiCrAlYZr-Cr_3_C_2_-ZrB_2_, and CoNiCrAlY-Cr_3_C_2_-Y_2_O_3_,	-	NiCrAlY + Y_2_O_3_ has lowest Mn content of 15.1 ± 0.6%	Hearth rolls	[51]
High speed flame spray	WC-Cr_3_C_2_Ni	590–890	Life of rolls improved to 3 times of conventional chrome plated rolls	Process rolls	[52]
Gas plasma spray	WC cermet		Prevent dents ad extend the life of by 5 times of chrome plated rolls	Conductor rolls	[52]
Flame spray	WC cermet		Developed fine coating by adding carbides, extend life by 3 times	Deflector rolls	[52]
Flame spray	MCrAlY		Reducing the Al content is effective to prevent Mn build up	Hearth rolls	[52]
Detonation gun spray	WC cermet	500–700	Service life is extended from 5 to 25 months and corrosion rate reduced from 35 g/m^2^h to 1 g/m^2^h	Bridle rolls	[53]
Plasma spray	WC self fluxing alloy		Service life is extended from < 2 months to > 25 months and corrosion rate reduced from 35 g/m^2^h to 1 g/m^2^h	Conductor rolls	[53]

Note: APS = atmospheric plasma spray, SAPS = supersonic atmospheric plasma spray, HVOF = high velocity oxygen fuel, SEN = submerged entry nozzle.

**Table 2:** Thermal spray processes and feedstock materials that have replaced hard chrome plating.

Thermal Spray Process	Feedstock Materials	Analysed Based on	Comments	References
HVOF	WC-Co and Tribaloy 400	Fatigue life	Hardness improved to 12.8 GPa compared to hard chrome 10.1 GPa	[81]
HVAF	WC-Co-Cr	Fatigue life at extreme temperature and compressive force environments	Coatings exhibited a fatigue life more than 10e7 cycles. Recommended a layer thickness of 65–80 µm instead of 250 µm	[82]
Laser cladding	Ni-WC	Wear and bending strength	Outperformed hard chrome plating with respect to wear and hardness. Coating could withstand more than twice the bending tool displacement than the chrome electroplating	[83]
Chemical vapour deposition (CVD)	(Hardide-A) WC/W metal matrix composite	Wear, high temperature oxidation, abrasion, impact and fatigue resistance	Outperformed hard chrome plating in all aspects	[84]
HVOF	Cr_3_C_2_-NiCr	High temperature oxidation and wear resistant	Outperformed hard chrome plating in all aspects	[85]
Physical vapour deposition (PVD)	Metal and diamond-like carbon to create BALINIT C, a WC/C coating	High temperature oxidation and wear resistant	High hardness between 1000 and 1500 HV. Lower coefficient of friction (0.1–0.2 against steel, dry) compared to hard chrome plating (approx. 0.5).	[86]

**Table 3:** Development of coating material and thermal spray process to produce thermal barrier coatings for the iron and steel making hardware.

Process	Coating Material	Target	Comments	Ref.
APS and laser glazing	CYSZ	Thermal life cycle	Lifetime of CYSZ coatings was improved with average of failure cycles number for as sprayed and laser glazed coating was 253 and 309 cycles	[9]
APS	YSZ	Thermal shock resistance	Minimum life 1.1 × 10^4^ h at 1500 K	[10]
Plasma jet and oxy-acetylene flame spray	Zirconia or alumina base cermet layer 60–62 wt% of Ni, 12–15 wt% Cr, plus Fe, Mn, and C	Thermal shock resistance	Average operation time of blast-furnace tuyere increased from 4 months to 6 months. Excellent mechanical strength, antioxidation and thermal-shock resistance > 1000 °C	[22]
EB-PVD and APS	YPSZ top coat and a NiCoCrAlY/PtAl-based metallic bond coat	Thermal cycle life and thermal conductivity	EB-PVD YPSZ top coats sustained 400 thermal cycles at 1150 °C compared 250 cycles of APS YPSZ.	[26]
APS	Gd_2_Zr_2_O_7_/YSZ (GZ/YSZ) and La_2_Zr_2_O_7_/YSZ ((LZ/YSZ)	Thermal shock resistance	GZ/YSZ exhibited superior thermal shock resistance to the LZ/YSZ due to its high K_IC_/E value of 21 × 10^−6^ m^1/2^	[90]
APS	GZ, GZ/YSZ (prepared by mixed powder of Gd_2_Zr_2_O_7_ and YSZ), and GSZC (prepared by (Gd_0.925_Sc_0.075_)_2_(Zr0.7Ce_0.3_)_2_O_7_ powder)	Thermal shock resistance	GZ coatings sustained for the longer time (38 cycles) compared to other two coatings (33 cycles for GZ/YSZ and 7 cycles for GSZC) in thermal shock tests between 900 °C and 1450 °C temperatures	[91]
APS	Stoichiometric (La_0.8_Gd_0.2_)_2_Ce_2_O_7_ (LGC) and Double-ceramic-layer (DCL) optimum (La_0.8_Gd_0.2_)_2_Ce_2_O_7_/YSZ (LGC/YSZ)	Thermal shock resistance	LGC/YSZ (DCL) TBCs had better thermal shock resistance ability than that of LGC TBCs, which was ~109 cycles at 1100 °C.	[93]
APS and laser-glazed	Functionally graded (FG) lanthanum magnesium hexaluminate (LaMgAl_11_O_19_)/YSZ and dual layer (LaMgAl_11_O_19_)/YSZ (DC-TBC) TBCs	Thermal shock resistance and thermal insulation capability	Laser glazed FG-TBCs sustained 170 thermal cycles compared to 90 cycles of laser glazed DC-TBC. Laser glazing improved the thermal cycles of FG-TBCs from 100 to 170 cycles.	[94]
APS	NiCrAlY bond coat, and nanostructured and conventional YSZ topcoats	Bonding strength and thermal insulation capability	Adhesion strength of nanostructured TBC was 38.21 MPa, improved from 25.35 MPa of the conventional YSZ TBC	[95]

## Data Availability

No new data were created or analyzed in this study. Data sharing is not applicable to this article.
